# Hypermethylation of mismatch repair gene *hMSH2* associates with platinum-resistant disease in epithelial ovarian cancer

**DOI:** 10.1186/s13148-019-0748-4

**Published:** 2019-10-30

**Authors:** Hua Tian, Li Yan, Li Xiao-fei, Sun Hai-yan, Chen Juan, Kang Shan

**Affiliations:** 1 Department of Molecular Biology, Hebei Medical University, Fourth Hospital, Shijiazhuang, China; 2 Department of Obstetrics and Gynaecology, Affiliated Xing Tai People Hospital of Hebei Medial University, Xingtai, China; 3 Department of Obstetrics and Gynaecology, Hebei Medical University, Fourth Hospital, Shijiazhuang, China

**Keywords:** *hMSH2*, Mismatch repair, DNA methylation, RRBS, Prognosis

## Abstract

**Purpose:**

One major reason of the high mortality of epithelial ovarian cancer (EOC) is due to platinum-based chemotherapy resistance. Aberrant DNA methylation may be a potential mechanism underlying the development of platinum resistance in EOC. The purpose of this study is to discover potential aberrant DNA methylation that contributes to drug resistance.

**Methods:**

By initially screening of 16 platinum-sensitive/resistant samples from EOC patients with reduced representation bisulfite sequencing (RRBS), the upstream region of the *hMSH2* gene was discovered hypermethylated in the platinum-resistant group. The effect of *hMSH2* methylation on the cellular response to cisplatin was explored by demethylation and knockdown assays in ovarian cancer cell line A2780. Matrix-assisted laser desorption ionization time-of-flight (MALDI-TOF) mass spectrometry was employed to examine the methylation levels of *hMSH2* upstream region in additional 40 EOC patient samples. RT-qPCR and IHC assay was used to detect the *hMSH2* mRNA and protein expression in extended 150 patients.

**Results:**

RRBS assay discovered an upstream region from − 1193 to − 1125 of *hMSH2* was significant hypermethylated in resistant EOC patients (*P* = 1.06 × 10^−14^). In vitro analysis demonstrated that global demethylation increased cisplatin sensitivity along with a higher expression of the hMSH2 mRNA and protein. Knockdown *hMSH2* reduced the cell sensitivity to cisplatin. MALDI-TOF mass spectrometry assay validated the strong association of hypermethylation of *hMSH2* upstream region with platinum resistance. Spearman’s correlation analysis revealed a significantly negative connection between methylation level of *hMSH2* upstream region and its expression. The Kaplan-Meier analyses showed the high methylation of *hMSH2* promoter region, and its low expressions are associated with worse survival. In multivariable models, *hMSH2* low expression was an independent factor predicting poor outcome (*P* = 0.03, HR = 1.91, 95%CI = 1.85–2.31).

**Conclusion:**

The hypermethylation of *hMSH2* upstream region is associated with platinum resistant in EOC, and low expression of *hMSH2* may be an index for the poor prognosis.

## Introduction

In the female reproductive system, epithelial ovarian cancer (EOC) is the third most common cancer and the first leading cause of cancer deaths [1]. Main reasons for the high mortality include late diagnosis and drug resistance [2]. Currently, the standard treatment is platinum-based chemotherapy following primary debulking surgery for advanced patients. However, approximately 20% of patients fail to respond to platinum-based chemotherapy [3], and up to 75% of patients among the initial responders eventually relapse within less than 2 years [4]. Accordingly, primary or acquired resistance to chemotherapeutic drugs is a major obstacle in the treatment of ovarian cancer and the main contributing factor for the cancerous death.

Increasing evidence also has shown that epigenetic changes may play an important role in chemotherapy resistance of ovarian cancer [5]. In particular, it has been suggested that DNA methylation, a well-studied epigenetic change, may serve as a potential biomarker for chemotherapy-resistant phenotypic screening [6]. Nevertheless, the more detailed mechanism of how DNA methylation affects the drug-resistant still needs to be explored. We used reduced representation bisulfite sequencing (RRBS) screened out a significantly hypermethylated upstream region of *hMSH2*, which involved in DNA mismatch repair (MMR) system. In mammals, MMR genes play a key role in not only DNA replication and repair [7] but also DNA damage signals and consequent apoptosis [8]. Because chemotherapy is a mainstay of DNA-damaging agents in numerous cancer therapies, loss of MMR proteins renders cells resistant to DNA-damaging regents [9, 10]. Interestingly, the existing studies about *hMSH2* showed inconsistent opinions on whether loss of hMSH2 expression can lead to resistance of cisplatin or not. Early studies using immunohistochemical staining with tumor sections suggested hMSH2 expression was not highly predictive of drug sensitivity as measured by response, progression-free survival (PFS), or overall survival (OS) [11, 12]. However, a recent study using whole-genome CRISPR (clustered regularly interspaced short palindromic repeats) screen in a bladder cancer cell line identified that *hMSH2* was the most significantly enriched gene that promotes resistance to cisplatin [13]. In addition to genetic mutations, promoter hypermethylation is an important mechanism for the loss of *hMSH2* expression and has been reported to be associated with some human cancers [14, 15]. In ovarian cancer, the methylation frequency of *hMSH2* promoter has been reported to be as high as 51.7%, and the methylation of *hMSH2* correlated with histological grade and lymphatic metastasis [16]. However, to date, there are no reports about the role of *hMSH2* expression loss caused by aberrant methylation of the promoter region in platinum resistance.

This study is to investigate the role of aberrant methylation of *hMSH2* upstream region involved in platinum resistance in EOC. Firstly, we have examined the possible role of higher expression of hMSH2 induced by global de-methylation and decreased expression by *hMSH2* knockdown on ovarian cancer cells to cisplatin. Further, we also examined the effects of methylation status and expression of hMSH2 in ovarian tumor samples on prognosis of EOC patients.

## Results

### Patient characteristics

Archived information of 150 EOC patients was obtained from the Hebei Medical University, Fourth Hospital. All patients received platinum-based chemotherapy following primary debulking surgery and followed up for 3 years at least. The median age of patients was 56 years old (age ranges from 20 to 78). In terms of histology, 85 (56.7%) out of the 150 patients were diagnosed with serous adenocarcinoma, 41 (27.3%) with endometrioid carcinoma, 9 (6.0%) with mucinous carcinoma, 6 (4.0%) with clear cell carcinoma, and 9 (6.0%) with mixed type. According to FIGO (International Federation of Gynecology and Obstetrics) staging, 112 cases (81.3%) had stage III–IV ovarian cancer and 28 cases (18.7%) in stages I–II. Histologically, 38 (25.3%) tumors were G1 grade, 67 (44.7%) were G2 grade, and 45 (30.0%) were G3 grade. Detailed information was shown in Table 1.

**Table 1:** Patient information and dosimetric parameters

Characters	Histology/stage	Patients (*n*)	Median	Percentage/range
Age			56 years	20–78 years
Histology	Serous	85		56.7%
	Endometrioid	41		27.3%
	Mucinous	9		6.0%
	Clear cell	6		4.0%
	Mixed type	9		6.0%
FIGO stage	I–II	28		18.7%
	III–IV	122		81.3%
Histological grade	G1	38		25.3%
	G2	67		44.7%
	G3	45		30.0%
Tumor residual size	0 cm	42		28.0%
	≤ 1 cm	71		47.3%
	> 1 cm	37		24.7%
Tumor size	≤ 10 cm	67		44.7%
	> 10 cm	83		55.3%
Platinum-based	Cisplatin-based	31		20.7%
	Carboplatin-based	119		79.3%
Follow-up time		150	36.5 months	2–86 months

*FIGO* International Federation of Gynecology and Obstetrics

### Screening with RRBS

Samples from 8 platinum-resistant and 8 platinum-sensitive EOC patients were screened using RRBS technique to identify differentially methylated loci between sets of samples. The detailed information of 16 patients was shown in Additional file 2: Table S1. In general, after removing the unqualified data, 276 valid hyper- or hypo-methylated regions were identified Additional file 3. We ranked these loci according to the *P* value of differential methylation regions (DMR) from lowest to highest and evaluated each site one by one to check whether there is any region that has a potential connection with the drug-resistant. Most loci on the top of the list were from transcript factors or regions with unknown function. Notably, among the non-transcript factor gene-related loci, an upstream region from − 1193 to − 1125 of the *hMSH2* gene was identified with a significant DMR *P* value of 1.06 × 10^−14^, meaning the methylation status of *hMSH2* was thought to be putatively related to EOC patients’ response to platinum agents.

### Global demethylation and expression changing of *hMSH2* in A2780 cells

**Fig. 1:**
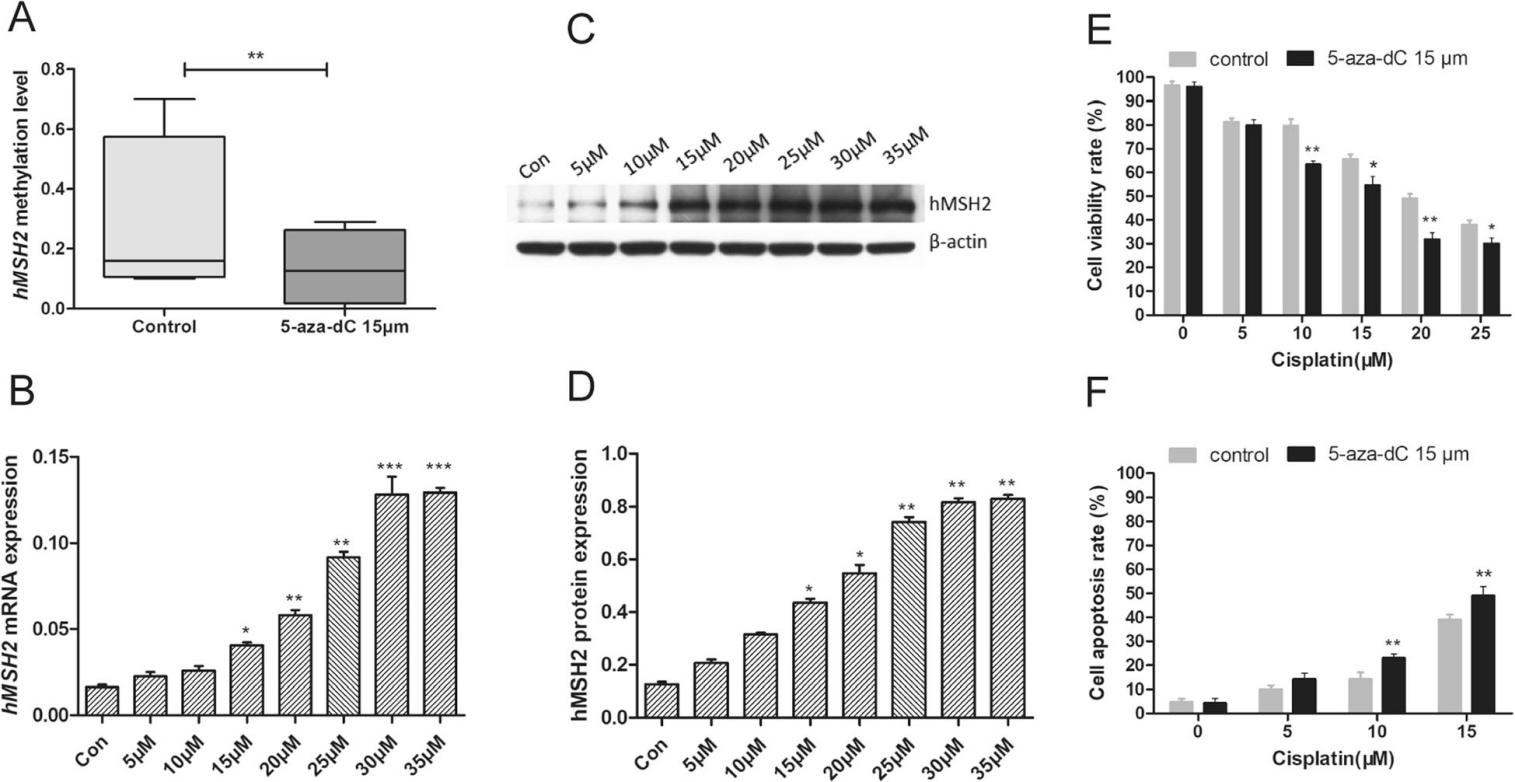
Effects of 5-aza-dC treatment on the ovarian cancer cellular sensitivity to cisplatin. **a** The alterations of the methylation level of *hMSH2* promoter in A2780 cells after 5-aza-dC treatment (15 μM) for 72 h. **b** The change of *hMSH2* mRNA expression after treated with the increasing concentrations of 5-aza-dC for 72 h. **c**, **d** Western bolt assay showed the increase trend of hMSH2 protein in A2780 cells after treated with the increasing concentrations of 5-aza-dC for 72 h. **e** CCK-8 assays suggested A2780 cells pre-treated with 5-aza-dC (15 μM) for 72 h showed the obvious decreased proliferation rates to cisplatin compared to control cells (DMSO treatment). **f** The cell apoptosis rates were significantly increased in A2780 pre-treated with 15 μM 5-aza-dC for 72 h than that in control cells (DMSO treatment) at several cisplatin concentrations by flow cytometry. *P < 0.05; **P < 0.01; ***P < 0.001. Each assay was performed in triplicate

Since in vitro evidence has shown that *hMSH2* was the most enriched gene for the cisplatin resistant [13] and it also has been widely accepted that hypermethylation at promoter regions can suppress the gene expression; therefore, our hypothesis is that the hypermethylation of promoter for the *hMSH2* might account for the drug resistance. We first tested our hypothesis in A2780 cells, an ovarian cancer cell line that was established from tumor tissue from an untreated patient. The A2780 cells were treated with the 5-aza-dC, a global demethylation reagent, and the methylation of the *hMSH2* upstream region was assessed with MALDI-TOF mass spectrometry. The mass spectrometry results showed that the methylation level of the *hMSH2* upstream region was remarkably reduced by 1.9-fold by average after the administration of 15 μM 5-aza-dC for 72 h (*P* < 0.05, Fig. 1a). Furthermore, RT-qPCR and western blot assays both demonstrated a significant increase of hMSH2 expression following treatment with 5-aza-dC 15 μM (*P* < 0.05, Fig. 1b–d). More importantly, the hMSH2 expression level was positively correlated with the increasing concentrations of 5-aza-dC, which indicated a direct association of hMSH2 expression with the methylation.

### Demethylation of *hMSH2* enhances sensitivity to cisplatin in A2780 cells

Cell viability and apoptosis assays were performed to determine the effect of *hMSH2* demethylation in A2780 cells on cisplatin sensitivity. A2780 cells that had been pre-treated with 15 μM 5-aza-dC were exposed to 0, 5, 10, 15, 20, and 25 μM cisplatin, respectively, for 24 h. The control cells were pre-treated with DMSO. As shown in Fig. 1e, the cell viability rates of 5-aza-dC treatment groups showed obviously decreased compared with control groups. By flow cytometry, the cisplatin-induced apoptosis rates were remarkably increased in 5-aza-dC treatment groups compared with the control groups (*P* < 0.05, Fig. 1f).

### Suppression of hMSH2 increases cisplatin resistance in A2780 cells

**Fig. 2:**
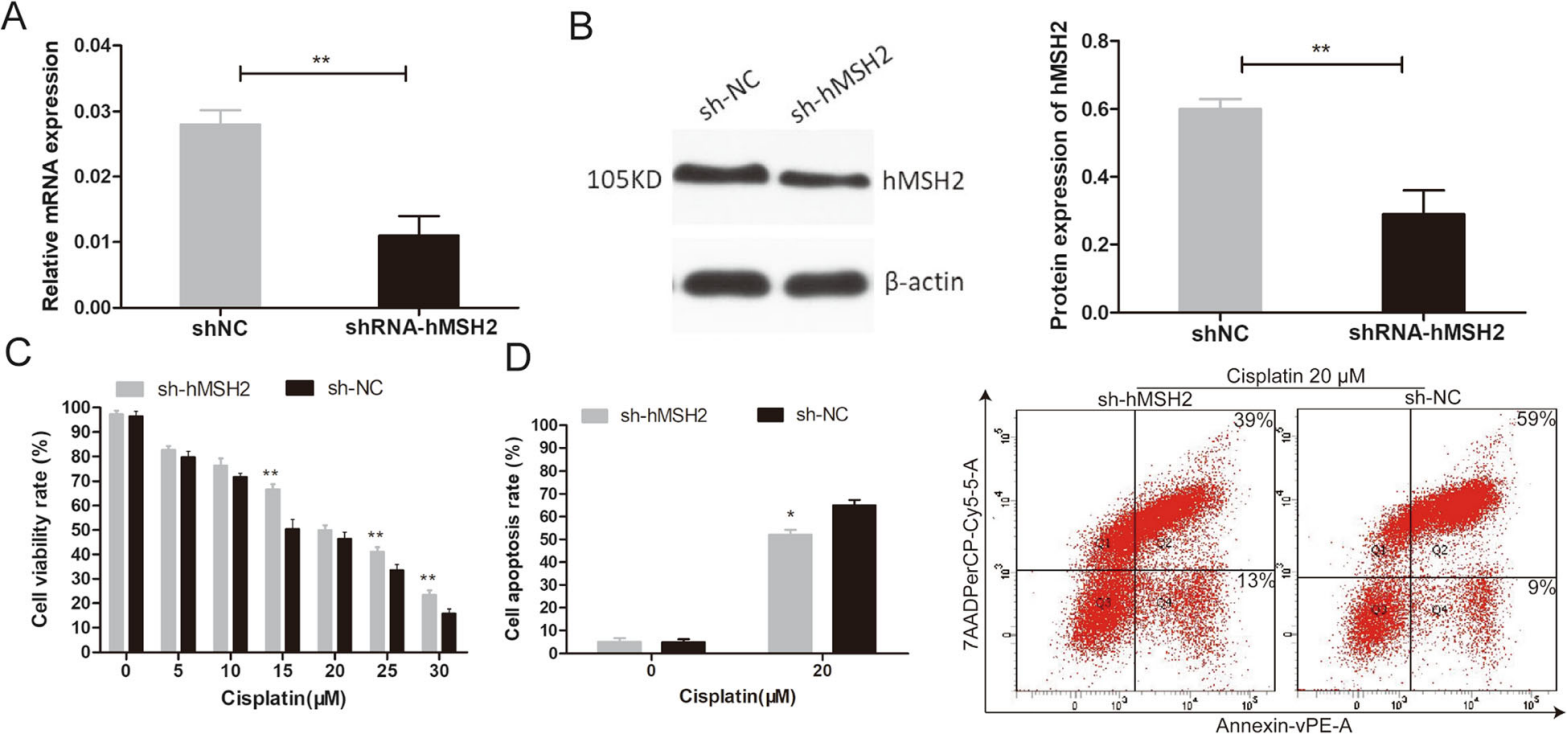
The alteration of the sensitivity to cisplatin after a knockdown of *hMSH2* expression in A2780 cells. **a**, **b** RT-qPCR and western blot assay showed the reduced expression of hMSH2 in sh-hMSH2 cells compared to shNC cells. **c** CCK-8 assays showed a significant increase in the proliferation rates in the shRNA-hMSH2 cells compared with the shNC cells after cisplatin treatment at several concentrations for 24 h. **d** Flow cytometry showed that the apoptosis rate in the shRNA-hMSH2 cells was significantly lower than that in the shNC cells after exposure to cisplatin at the 20 μM concentration for 24 h. The A2780 cell apoptosis rates in each group. *P < 0.05; **P < 0.01. The experiments were repeated three times

To further affirm that the expression level of hMSH2 can independently affect the sensitivity of cisplatin, we knocked down the expression of *hMSH2* in A2780 cells via shRNA. *hMSH2* mRNA and protein expression levels were reduced by 63% and 58% in the A2780 shRNA-hMSH2 group, respectively, compared with the A2780 shNC group (*P* < 0.05, Fig. 2a, b).

Then, we compared the cisplatin sensitivity of *hMSH2* knockdown cells and control cells. CCK-8 assays showed a significant increase in the proliferation rates in the shRNA-hMSH2 group compared with the shNC group after cisplatin treatment at several concentrations for 24 h (*P* < 0.05, Fig. 2c). Next, flow cytometry further confirmed that the apoptosis rate in the shRNA-hMSH2 group was significantly lower than that in the shNC group after exposure to cisplatin at 20 μM concentration (*P* = 0.03, Fig. 2d).

### Association between *hMSH2* promoter methylation level and platinum resistance of EOC patients

**Fig. 3:**
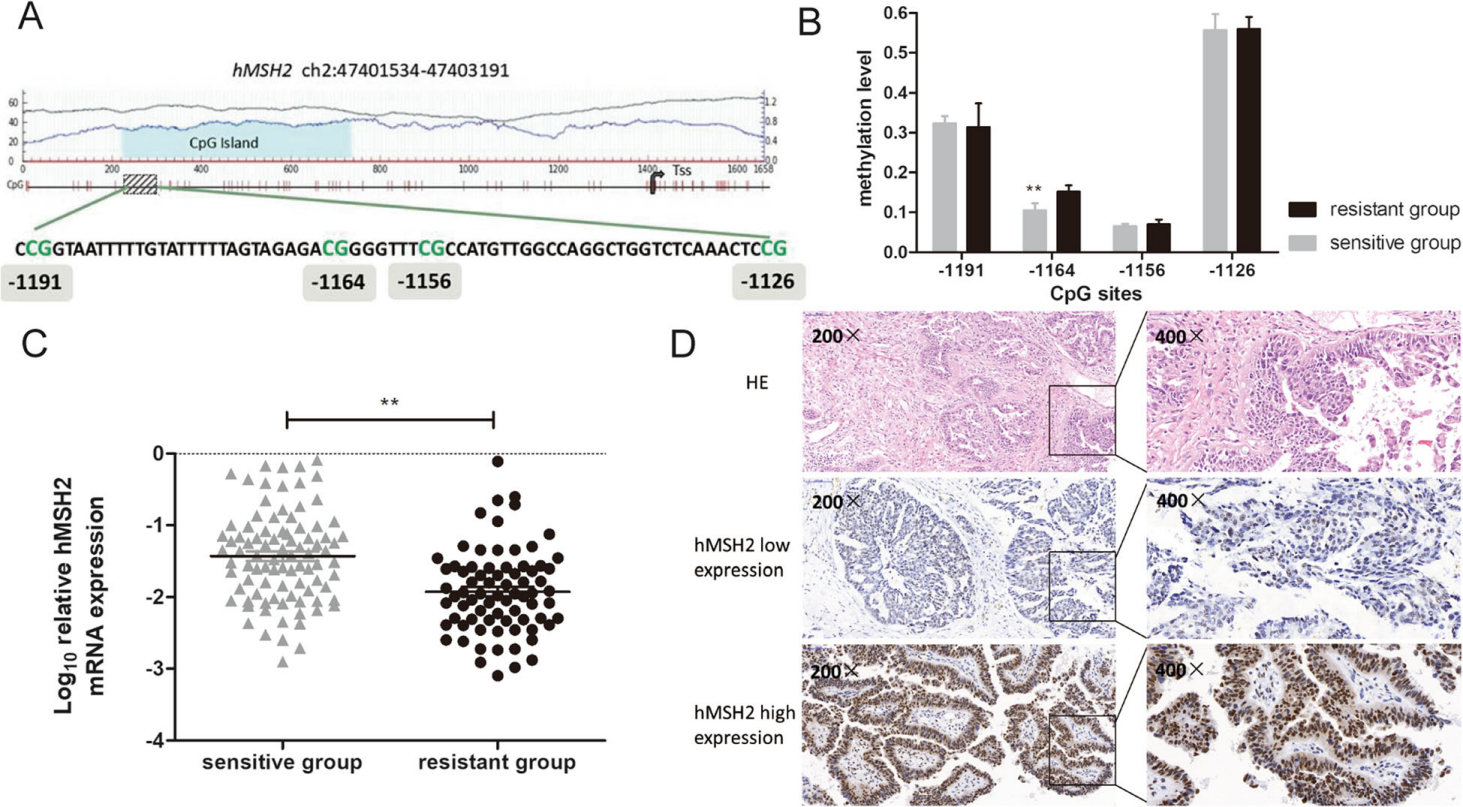
The methylation level of *hMSH2* promoter was associated with platinum-resistant in EOC patients. **a** Reduced representation bisulfite sequencing (RRBS) assay showed the region (− 1193 to − 1125 upstream) within the promoter of hMSH2 was hypermethylated in platinum-resistant patients compared with platinum-sensitive patients (*P* = 1.06 × 10^−14^). **b** The methylation of − 1164 CpG site was significantly hypermethylated in platinum-resistant patients compared with platinum-sensitive patients by MALDI-TOF mass. **c** The mRNA expression of *hMSH2* in platinum-resistant patients and platinum-sensitive patients. All experiments were repeated three times. **d** Images shown the expression of hMSH2 protein in EOC tumor tissues. *P < 0.05; **P < 0.01

The previous cell studies suggested that hMSH2 expression can independently account for the cell susceptibility to cisplatin, and the expression level of hMSH2 was affected by the methylation. Therefore, we hypothesized that *hMSH2* promoter might be differentially methylated and thus accounts for varieties of drug sensibility in EOC patients. In the previous results, the RRBS assay has demonstrated that the upstream region from − 1193 to − 1125 of the *hMSH2* gene was significantly hypermethylated in the platinum-resistant group (Fig. 3a). To further confirm the findings of the RRBS assay, MALDI-TOF mass spectrometry was employed to examine the methylation level of this region in an independent group of samples that were from 18 platinum-resistant EOC patients and 22 platinum-sensitive EOC patients. The analysis from MALDI-TOF mass spectrometry revealed that the methylation level of the − 1164 CpG site was significantly higher in the platinum-resistant group than that in the platinum-sensitive group (*P* = 0.004, Fig. 3b). However, the methylation levels of the − 1191, − 1156, and − 1126 CpG sites were not significantly different between the two groups (all *P* > 0.05) (Additional file 3).

### Correlation analysis of *hMSH2* up-stream methylation status and its expression in EOC patients

RT-qPCR was used to detect the mRNA levels of *hMSH2* in an extended sample group of 60 platinum-resistant EOC patients and 90 platinum-sensitive EOC patients. The *hMSH2* mRNA level in the platinum-resistant group was 1.95-fold lower than that in the platinum-sensitive group, and the difference was statistically significant (*P* = 0.004, Fig. 3c). Spearman’s correlation analysis revealed that there was a significant negative connection between the methylation level of the *hMSH2* promoter and its mRNA expression (*P* = 0.02, *r* = −.41). The result demonstrated that the hypermethylation of *hMSH2* promoter may be responsible for the downregulation of its expression in EOC tumor tissues.

Further, among the 150 patient samples, IHC analysis was conducted to examine the protein expression of hMSH2 in 37 platinum-resistant EOC patients and 49 platinum-sensitive EOC patients. Compared with the platinum-sensitive group, the expression of the hMSH2 protein was significantly decreased in the platinum-resistant group (*P* = 0.03, Table 2). Representative images of hMSH2 staining are shown in Fig. 3d.

**Table 2:** Associations of platinum-based chemotherapy resistance with hMSH2 protein expression

hMSH2 expression	Resistant group, *n* (%)	Sensitive group, *n* (%)	*P*
High	21 (56.75)	38 (77.55)	0.03
Low	16 (43.24)	11 (22.45)	

### High methylation of *hMSH2*promoter region correlates with poor EOC patients’ prognosis

**Fig. 4:**
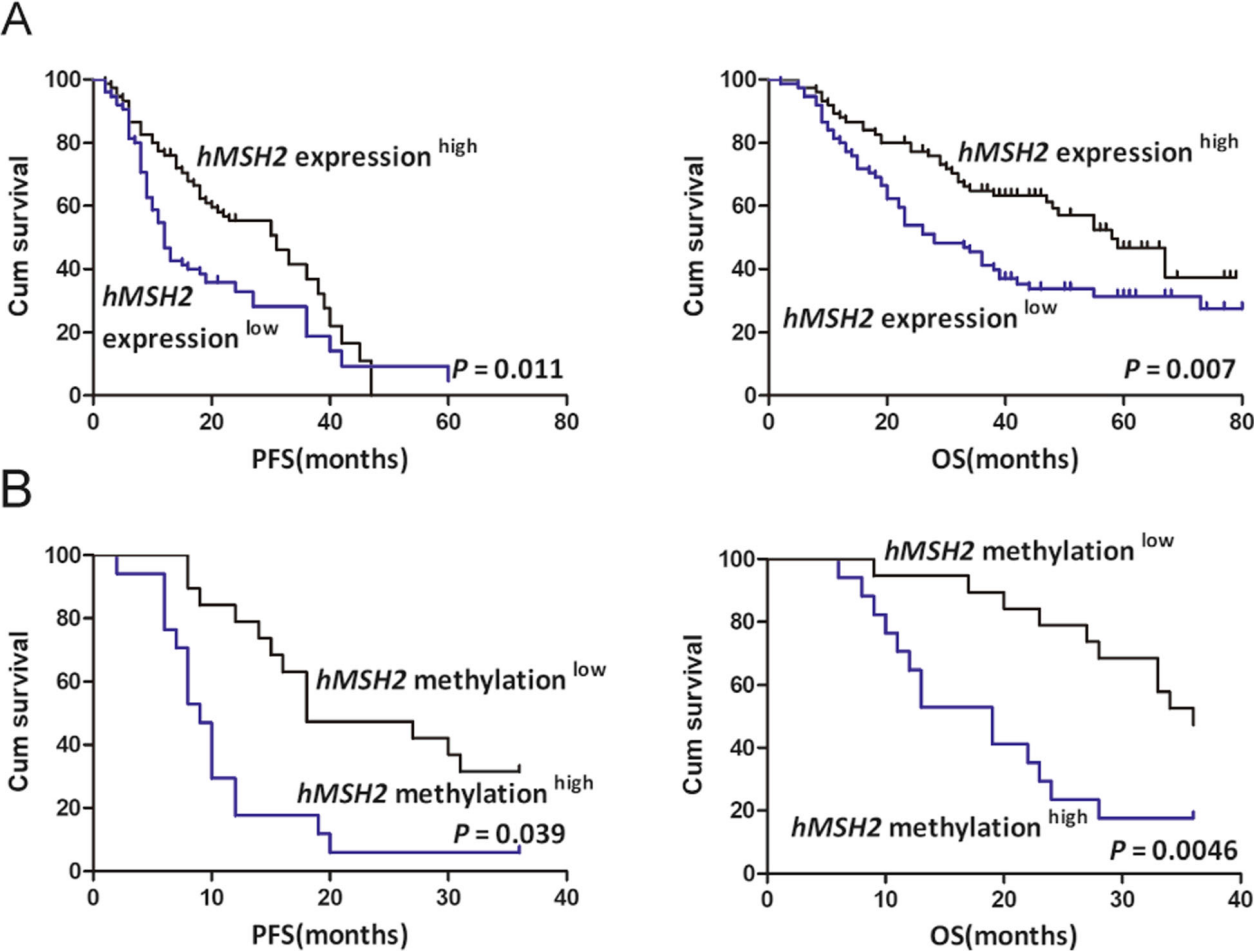
The high methylation of *hMSH2* and its low mRNA expression are associated with poor survival in EOC patients. **a** Kaplan-Meier analysis of 150 EOC patients’ survival according to the *hMSH2* expression. **b** Kaplan-Meier analysis of PFS and OS according to the *hMSH2* methylation level in 40 EOC patients

Previously described methylation of *hMSH2* and its expression were divided into low and high groups based on their median value, respectively. Kaplan-Meier analysis demonstrated that patients in the *hMSH2* methylation^high^ group had shorter PFS and OS than those in *hMSH2* methylation^low^ group, and *hMSH2* expression^low^ group was associated with poorer prognosis compared to *hMSH2* expression^high^ group (Fig. 4a, b).

Based on the negative correlation between methylation status of *hMSH2* upstream region and its expression in EOC patients in this study, *hMSH2* expression index was included in multivariable analysis for survival. The results showed the *hMSH2* mRNA levels were significantly associated with OS when age, stage, grade, tumor size, and tumor residual size were included in the model (*P* = 0.03, HR = 1.91, 95%CI = 1.85–2.31, Table 3), but there was no obvious relationship between *hMSH2* mRNA expression and PFS (*P* = 0.11, Table 3). These analyses indicated that *hMSH2* mRNA expression might serve as an independent prognostic biomarker for EOC patients.

**Table 3:** Prognostic factors in epithelial ovarian cancer patients using the Cox proportional hazards model

	OS				PFS	
	HR	(95%CI)	*P*	HR	(95%CI)	*P*
Age						
≤ 50 vs > 50	0.988	0.63–1.56	0.96	1.14	0.74–1.75	0.54
FIGO stage						
I–II vs III–IV	0.890	1.14–5.33	0.01	1.12	1.22–6.65	0.04
Grade						
G1–2 vs G3	0.992	0.60–1.63	0.97	0.78	0.49–1.23	0.29
Tumor residual size						
0 cm vs ≤ 1 cm vs > 1 cm	0.189	0.09–0.36	0.00	0.24	0.14–0.41	0.00
Tumor size						
≤ 10 cm vs > 10 cm	1.564	0.78–1.56	0.57	0.89	0.89–2.86	0.67
*hMSH2* expression						
Low vs high	1.916	1.85–2.31	*0.03*	1.43	0.92–2.20	0.11

*FIGO* International Federation of Gynecology and Obstetrics

## Discussion

It has been suggested that DNA methylation-induced silencing of various drug response genes and pathways may facilitate the development of drug resistance in ovarian cancer. The present study revealed that (1) 5-aza-dC-induced demethylation can significantly increase the sensitivity of ovarian cancer cells to cisplatin as well as *hMSH2* expression, (2) knockdown of *hMSH2* expression could desensitize A2780 ovarian cancer cells to cisplatin, (3) the *hMSH2* upstream region was significantly hypermethylated in the EOC tissue of platinum-resistant patients, and (4) the lower expression of *hMSH2* due to hypermethylation of the upstream region was associated with the clinical outcome of patients with EOC. To the best of our knowledge, this is the first study to investigate the role of aberrant methylation of the *hMSH2* promoter region in EOC patient resistance to platinum-based chemotherapy.

Cisplatin or carboplatin in combination with paclitaxel is still the first-line chemotherapy drugs for advanced ovarian cancer. The chemotherapeutic mechanism of platinum compounds involves its covalent binding to DNA to form DNA adducts, which results in a DNA replication block and promotes cell death. hMSH2, by itself [17] or as an hMSH2-hMSH6 complex [18], recognizes specific DNA damage caused by cisplatin and carboplatin. In addition, hMSH2 can interact with ATR and recruit it to the sites of DNA damage, further activating a series of apoptosis proteins and resulting in apoptosis of the cells [19]. Therefore, *hMSH2* is considered to play an important role in the platinum resistance of ovarian cancer.

In a preliminary genome-wide methylation screen with samples from 8 platinum-resistant and 8 platinum-sensitive EOC patients, we discovered that the methylation level of *hMSH2* upstream region (− 1193 to − 1125) was significantly higher in the resistant group. In the following in vitro studies, we discovered that global demethylation could induce higher *hMSH2* mRNA and protein level as well as a correspondingly increased cisplatin sensitivity. Knockdown of the *hMSH2* expression alone can increase cell proliferation rates and decrease apoptosis rates when challenged with the cisplatin. A recent study in a breast cancer cell line also showed that the promoter hypermethylation-mediated inactivation of the *hMSH2* gene was associated with the acquired resistance against doxorubicin, and the demethylating agent 5-aza-dC and the HDAC inhibitor Trichostatin-A significantly re-sensitized resistant cells to doxorubicin [20]. Our findings are in line with this report, suggesting that the epigenetic inactivation of *hMSH2* could be one of the contributing factors for drug resistance. The therapeutic application of demethylation agents is attracting more attention to the treatment of EOC [21]. Demethylation of other genes at promoter regions has been shown to restore chemotherapeutical drug responses in cancer cells [22–24]. The result from this study suggested *hMSH2* might be another target of epigenetic therapy for EOC.

The mass spectrometry results further confirmed that − 1164 upstream region of *hMSH2*, a transcription factor Mef-2, HNFα, and GR binding site (http://jaspar.genereg.net/), was hypermethylated in patients with platinum resistance. We also discovered that the hMSH2 mRNA and protein levels were significantly downregulated in patients with platinum resistance. Correlation analysis suggested that the methylation level of − 1164 CpG site was associated with *hMSH2* mRNA expression. These findings indicated that loss of expression due to hypermethylation of *hMSH2* upstream region might induce platinum resistance in EOC patients. In the earlier literature hypermethylation of another essential MMR gene, *hMLH1* was observed in cisplatin-resistant EOC patients [25, 26]. While whether loss of *hMSH2* expression caused by hypermethylation of *hMSH2* promoter contributed to platinum resistance or not in EOC was unclear previously. The results from our study provided the evidence firstly that hypermethylation of the *hMSH2* upstream region could be another mechanism for the platinum resistance in EOC patients. Interestingly, a slight increase of *hMLH1* methylation level was also observed in the platinum-resistant patients compared to the sensitive patients in our study based on the RRBS analysis. However, the differences between the two groups were not statistically significant. Indeed, there are few pieces of previous literature reporting the correlation between *hMLH1* methylation and platinum resistance based on customized or commercially available methylation array or next-generation sequence (NGS)-based platforms. What is more, none of those studies reported a clear relationship between the *hMLH1* methylation and drug resistance [27–29]. Much more need to be done in the future.

Importantly, in this study, the Kaplan-Meier analyses showed the high methylation of *hMSH2* promoter region was associated with EOC patients’ worse survival. However, the obvious impact of *hMLH1* and *hMSH2* methylation on serous ovarian cancer patients’ survival was not observed in TCGA dataset (Additional file 1: Figure S1). We carefully examined the dataset and discovered that there were many heterogeneity of the patients provided by TCGA dataset, particularly too many varieties on the patients’ treatment plan. We believe this may be the key reason for the inconsistence between the TCGA result and our study. Further, multivariable analysis of 150 EOC patients showed that patients with lower *hMSH2* expression have a poorer prognosis than those with higher expression, which was also indicated the prognosis value of *hMSH2* methylation for EOC patients due to the negative correlation between *hMSH2* methylation and its expression.

## Conclusion

Our study demonstrated that *hMSH2* low expression due to hypermethylation may play an important role in platinum resistance in EOC and the expression profile of *hMSH2* could be a potential biomarker for the prognosis. *hMSH2* might be a target for epigenetic therapy in platinum-resistant patients. To the best of our knowledge, this study has the largest patient number for the drug resistance research involving *hMSH2*. Though a cohort with more patients is still needed in the future, our current work still helps clarify some previous inconsistent opinions and might benefit treatment plans.

## Materials and methods

### Cell culture

The human ovarian cancer cell line A2780 was purchased from iCell Bioscience Inc. (Shanghai, China) in January 2018. The cell line was authenticated by short tandem repeat (STR)-based profiling (Genetica DNA Laboratories Inc.) prior to purchase. A2780 cells were grown in RPMI 1640 medium (Gibco; Thermo Fisher Scientific, Inc.) containing 10% fetal bovine serum (Invitrogen Gibco, NY, USA) in a humidified atmosphere with 5% CO_2_ at 37 °C.

### 5-aza-2′-deoxycytidine treatment

A2780 cells were treated with the DNA demethylating agent 5-aza-2′-deoxycytidine (5-aza-dC, Sigma, St Louis, MO, USA) at different concentrations for 72 h, while the control cells were treated with dimethyl sulfoxide (DMSO). After the treatment, cells were collected for DNA, RNA, and protein isolation. The methylation levels of *hMSH2* were examined by MALDI-TOF mass spectrometry, and its expression was tested by RT-qPCR and western blot assay, respectively.

A2780 cells were exposed to different concentrations of cisplatin for 24 h, which had been pre-treated with 15 μM 5-aza-dC for 72 h prior to the cisplatin treatment. Afterward, cell viability and apoptosis assay were carried out in the post-treated cells.

### Cell transfection and cisplatin treatment

Short hairpin RNA (shRNA) of *hMSH2* was obtained from the Gene Pharmaceutical Technology Company (Shanghai, China). The designed four target sequences in the *hMSH2* gene were 5′-GCAGCAGTCAGAGCCCCTTAAC-3′(sh-hMSH2–1038); 5′-GCAGA ATTGAGGCAGACTTTA-3′(sh-hMSH2–1233); 5’GCTTTGCTCACGTGTCAAATG-3′(sh-hMSH2–1945) and 5′-GGGCTATATCAGAATACATTG-3′(sh-hMSH2–2416). The most effective construct, recombinant plasmid inserted with *hMSH2* gene shRNA expression vector PGPU6/GFP/Neo-hMSH2-1233 was selected for the study, while a random sequence of shRNA (shNC) was used as the negative control. A2780 cells were transfected with either shRNA-hMSH2 plasmid or shNC plasmid using the Lipofectamine™2000 transfection reagent (Invitrogen, USA) according to the manufacturer’s instructions. The mRNA and protein levels of hMSH2 were analyzed by RT-qPCR and western blot to confirm the transfection efficiency.

At 24 h after transfection, A2780 shRNA-hMSH2 and A2780 shNC were treated with different concentrations of cisplatin (Sigma-Aldrich, St., Louis, MO, USA) for 24 h. Thereafter, cell viability and apoptosis assays were performed to investigate the cellular response to cisplatin.

### Western blot assay

The collected cells were lysed in ice-cold RIPA buffer, and protein lysates were then quantified with a BCA Protein Assay Kit. Western blotting was carried out as described previously [24]. The following antibodies were purchased, as indicated: a primary antibody against hMSH2 (ab92473, Abcam, Cambridge, UK), β-Actin (ab8226, Abcam, Cambridge, UK), and an anti-rabbit secondary antibody (Rockland, Gilbertsville, PA, USA). β-Actin was employed as a loading control. Immunoreactive proteins were detected by an Odyssey infrared imaging system (LI-COR Biosciences, Lincoln, NE, USA), and band intensities were quantified using ImageJ.

### Cell viability assay

Cell viability was assessed using a standard Cell Counting Kit-8 solution (CCK-8) (MedChemExpress USA) assay according to the manufacturer’s instructions. Briefly, A2780 cells were seeded into 96-well plates. After treatment with drugs, 10 μL of CCK-8 reagent was added into every well and incubated for 3 h. The optical density was measured using a microplate reader (Thermo Fisher Scientific, Inc.) at 492 nm. Each experiment was repeated three times.

### Apoptosis assay

Apoptosis of A2780 cells was analyzed using an Annexin V Apoptosis Detection kit I (BD Biosciences, Franklin Lakes, NJ, USA). Briefly, cells were seeded into 6-well plates. After treatment with drugs, the adherent cells were trypsinized without EDTA and collected by centrifugation. After washing with PBS two times, the cells were resuspended in 100 μL of 1 × binding buffer and were subsequently incubated with 5 μL of Annexin V staining solution at room temperature for 30 min in the dark. Then, 400 μL of 1 × binding buffer was added, and the fluorescence intensity was evaluated on a FACS Aria™ (BD Biosciences) flow cytometer. Each assay was performed in triplicate.

### Tissue samples

Tissue samples were collected from 150 patients with histologically confirmed EOC in the Hebei Medical University, Fourth Hospital, between November 2011 and June 2015. Informed consent was obtained from each participant, and this study was approved by the Institute Medical Ethics Committee of the Hebei Medical University, Fourth Hospital.

The study participants were divided into a platinum-resistant group (*n* = 60) and a platinum-sensitive group (*n* = 90) based on a platinum-free interval (PFI), which was calculated from the date of the last platinum compound treatment to the date of disease progression. Patients with a PFI of less than 6 months are considered as platinum-resistant, whereas patients with a PFI of greater than 6 months are deemed platinum-sensitive [30]. All the participants were followed up for 3 years regularly. PFS and OS were used to evaluate the survival status of patients.

### DNA extraction and MALDI-TOF mass spectrometry

Of the 150 EOC tissue samples, 40 yielded high-quality DNA using the Wizard Genomic DNA Purification Kit (Promega, Madison, WI, USA) according to the manufacturer’s instructions. MALDI-TOF mass spectrometry (Sequenom, San Diego, California, USA), described by Breitling et al. [31], was performed for *hMSH2* methylation analysis by CapitalBio Co., Ltd. (Beijing, China).

### RNA extraction and real-time quantitative PCR

Total RNA was isolated from 150 EOC tissue samples using TRIZOL reagent (Generay Biotech, Co., Ltd., Shanghai, China) according to the manufacturer’s protocols. The cDNA was then synthesized using a Revert Aid First Strand cDNA Synthesis Kit (Thermo Scientific, USA). The PCRs were carried out using a QuantiNova TMSYBR® Green PCR Kit (Qiagen, Hilden, Germany). Relative expression levels of *hMSH2* were calculated with the 2^–ΔCt^ method using *GAPDH* as an endogenous control, and all experiments were repeated three times.

### Immunohistochemistry

Of the 150 patients, 86 paraffin-embedded EOC tissue samples obtained from the pathology department of Hebei Medical University, Fourth Hospital, were used for immunohistochemistry (IHC) staining of hMSH2. Immunohistochemical reactions were performed using anti-hMSH2 antibody (ab52266, Abcam, Cambridge, UK, dilution, 1:2000). The judgment criteria for the IHC results were that hMSH2 protein was located in the nucleus, as evaluated according to the percentage of positive staining area and the staining intensity. A sum of the 2 items ≥ 4 was defined as high expression, and a sum < 4 was defined as low expression [32]. All analyses were conducted in a double-blind manner.

### Statistical analysis

All statistical analyses were performed using SPSS 21.0 statistical software package (Chicago, IL, USA). *P* value < 0.05 was considered statistically significant. Data from cell viability assay and cell apoptosis assay were analyzed by *t* test. The comparisons of *hMSH2* methylation levels and its mRNA expression between the two groups were carried out using the Wilcoxon Rank Sum test. The *χ*^2^ test was conducted to compare hMSH2 protein expression between groups. Spearman’s correlation analysis was used to evaluate the relationship between hMSH2 expression and methylation status. Kaplan-Meier analysis and Cox proportional hazard model were performed to analyze the association of *hMSH2* methylation and its expression with EOC patients’ prognosis.

## Supplementary information

**Additional file 1: Figure S1.** The impact of *hMLH1* and *hMSH2* methylation on serous ovarian cancer patients’ survival was presented from the TCGA dataset. (A-B) Kaplan-Meier analysis of PFS and OS according to the *hMLH1* and *hMSH2* methylation level in 496 serous ovarian cancer patients.

**Additional file 2: Table S1.** Clinicopathological information for the discovery cohort patients with ovarian cancer.

**Additional file 3: Table S2.** Thestatistics analysis data of 276 genes with differential methylation regions between platinum-resistant EOC tissues and platinum-sensitive EOC tissues through RRBS assay.

## Data Availability

The protocols are detailed in the manuscript for scientists wishing to use them for their research work. Also, the supporting data will be made available to editors and peer-reviewers, if required for the purposes of evaluating the manuscript.

## Abbreviations

5-aza-dC: 5-aza-2′-deoxycytidine
CCK-8: Cell counting kit-8 solution
DMR: Differential methylation region
EOC: Epithelial ovarian cancer
hMSH2: Human MutS homolog 2
IHC: Immunohistochemical
MMR: Mismatch repair
OS: Overall survival
PFI: Platinum-free interval
PFS: Progression-free survival
RRBS: Reduced representation bisulfite sequencing

## References

[1] Siegel RL, Miller KD, Jemal A (2017). Cancer statistics, 2018. Ca A Cancer J Clin.

[2] Lengyel E (2010). Ovarian cancer development and metastasis. Am J Pathol.

[3] Cannistra SA (2004). Cancer of the ovary. N Engl J Med.

[4] Pignata S, Cecere CS, Du Bois A, Harter P, Heitz F (2017). Treatment of recurrent ovarian cancer. Ann Oncol.

[5] Borley J, Brown R (2015). Epigenetic mechanisms and therapeutic targets of chemotherapy resistance in epithelial ovarian cancer. Ann Med.

[6] Seeber LM, Van Diest PJ (2017). Epigenetics in ovarian cancer. Semin Cancer Biol.

[7] Sameer A, Nissar S, Fatima K (2014). Mismatch repair pathway: molecules, functions, and role in colorectal carcinogenesis. Eur J Cancer Prev.

[8] Martin LP, Hamilton TC, Schilder RJ (2008). Platinum resistance: the role of DNA repair pathways. Clin Cancer Res.

[9] Aebi S, Kurdi-Haidar B, Gordon R, Cenni B, Zheng H, Fink D, Christen R, Boland C, Koi M, Fishel R, Howell S (1996). Loss of DNA mismatch repair in acquired resistance to cisplatin. Cancer Res.

[10] CRR R, Silva MM, Quinet A, Cabral-Neto JB, CFM M (2018). DNA repair pathways and cisplatin resistance: an intimate relationship. Clinics.

[11] Samimi G, Fink D, Varki NM, Husain A, Hoskins WJ, Alberts DS, Howell SB (2000). Analysis of MLH1 and MSH2 expression in ovarian cancer before and after platinum drug-based chemotherapy. Clin Cancer Res.

[12] Magnowska M, Surowiak P, Nowakmarkwitz E, Michalak M, Magnowski P, Rokita W, Kedzia H, Zabel M, Spaczyński M (2008). Analysis of hMLH1 and hMSH2 expression in cisplatin-treated ovarian cancer patients. Ginekol Pol.

[13] Goodspeed A, Jean A, Costello JC (2019). A whole-genome CRISPR screen identifies a role of MSH2 in cisplatin-mediated cell death in muscle-invasive bladder cancer. Eur Urol.

[14] Shin KH, Shin JH, Kim JH, Park JG (2002). Mutational analysis of promoters of mismatch repair genes hMSH2 and hMLH1 in hereditary nonpolyposis colorectal cancer and early onset colorectal cancer patients: identification of three novel germ-line mutations in promoter of the hMSH2 gene. Cancer Res.

[15] Wang YC, Lu YP, Tseng RC, Lin RK, Chang JW, Chen JT, Shih CM, Chen CY (2003). Inactivation of hMLH1 and hMSH2 by promoter methylation in primary non-small cell lung tumors and matched sputum samples. J Clin Investig.

[16] Zhang H, Zhang S, Cui J, Zhang A, Shen L, Yu H (2010). Expression and promoter methylation status of mismatch repair gene hMLH1 and hMSH2 in epithelial ovarian cancer. Aust N Z J Obstet Gynaecol.

[17] Mello J, Acharya S, Fishel R, Essigmann J (1996). The mismatch-repair protein hMSH2 binds selectively to DNA adducts of the anticancer drug cisplatin. Chem Biol.

[18] Duckett D, Drummond J, Murchie A, Reardon J, Sancar A, Lilley D, Modrich P (1996). Human MutSalpha recognizes damaged DNA base pairs containing O6-methylguanine, O4-methylthymine, or the cisplatin-d (GpG) adduct. Proc Natl Acad Sci U S A.

[19] Pabla N, Ma Z, McIlhatton M, Fishel R, Dong Z (2011). hMSH2 recruits ATR to DNA damage sites for activation during DNA damage-induced apoptosis. J Biol Chem.

[20] Ponnusamy L, Mahalingaiah PKS, Chang YW, Singh KP (2018). Reversal of epigenetic aberrations associated with the acquisition of doxorubicin resistance restores drug sensitivity in breast cancer cells. Eur J Pharm Sci.

[21] Matei D, Ghamande S, Roman LD, Alvarez SA, Nemunaitis J, Markham MJ, Nephew KP, Jueliger S, Oganesian A, Naim S (2018). A phase 1 clinical trial of guadecitabine and carboplatin in platinum-resistant, recurrent ovarian cancer: clinical, pharmacokinetic and pharmacodynamic analyses. Clin Cancer Res.

[22] Balch C, Yan P, Craft T, Young S, Skalnik DG, Huang TH, Nephew KP (2005). Antimitogenic and chemosensitizing effects of the methylation inhibitor zebularine in ovarian cancer. Mol Cancer Ther.

[23] Li Y, Hu W, Shen DY, Kavanagh JJ, Fu S (2009). Azacitidine enhances sensitivity of platinum-resistant ovarian cancer cells to carboplatin through induction of apoptosis. Am J Obstet Gynecol.

[24] You S, Kim M, Gupta A, Park MH, Weisenberger DJ, Liang G, Kim J (2018). Rewiring of cisplatin-resistant bladder cancer cells through epigenetic regulation of genes involved in amino acid metabolism. Theranostics.

[25] Strathdee G, Mackean M, Brown R (1999). A role for methylation of the hMLH1 promoter in loss of hMLH1 expression and drug resistance in ovarian cancer. Oncogene.

[26] Yan B, Yin F, Wang QI, Zhang W, Li LI (2016). Integration and bioinformatics analysis of DNA-methylated genes associated with drug resistance in ovarian cancer. Oncol Lett.

[27] Leon MD, Cardenas H, Vieth E, Segar M, Liu Y, Nephew K, Matei D (2016). Transmembrane protein 88 (TMEM88) promoter hypomethylation is associated with platinum resistance in ovarian cancer. Gynecol Oncol.

[28] Lum E, Vigliotti M, Banerjee N, Cutter N, Wrzeszczynski KO, Khan S, Kamalakaran S, Levine DA, Dimitrova N, Lucito R (2013). Loss of DOK2 induces carboplatin resistance in ovarian cancer via suppression of apoptosis. Gynecol Oncol.

[29] Bauerschlag DO, Ammerpohl O, Bräutigam K, Schem C, Lin Q, Weigel MT, Hilpert F, Arnold N, Maass N, Meinhold-Heerlein I (2011). Progression-free survival in ovarian cancer is reflected in epigenetic DNA methylation profiles. Oncology.

[30] Armstrong D (2002). Relapsed ovarian cancer: challenges and management strategies for a chronic disease. Oncologist.

[31] Breitling LP, Yang R, Korn B, Burwinkel B, Brenner H (2011). Tobacco-smoking-related differential DNA methylation: 27K discovery and replication. Am J Hum Genet.

[32] Song Y, Zuo Y, Qian XL, Chen ZP, Wang SK, Song L, Peng LP (2017). Inhibition of microRNA-21-5p promotes the radiation sensitivity of non-small cell lung cancer through HMSH2. Cell Physiol Biochem.

