# Supplementary figures and images for: Hypermethylation of mismatch repair gene hMSH2 associates with platinum-resistant disease in epithelial ovarian cancer
Source: Clin Epigenetics. 2019 Oct 30;11:153. doi: 10.1186/s13148-019-0748-4 (PMC6822346; doi:10.1186/s13148-019-0748-4)

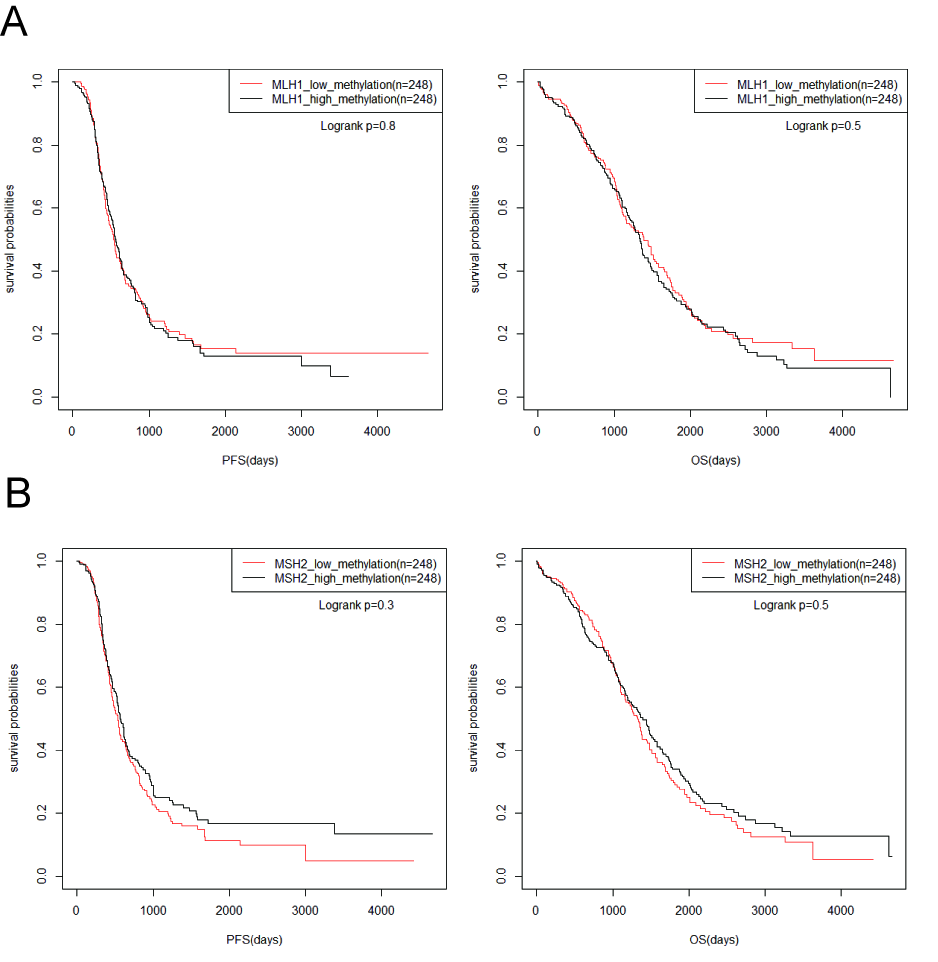

Supplement: Supplementary file 1 — Additional file 1: Figure S1. The impact of hMLH1 and hMSH2 methylation on serous ovarian cancer patients’ survival was presented from the TCGA dataset. (A-B) Kaplan-Meier analysis of PFS and OS according to the hMLH1 and hMSH2 methylation level in 496 serous ovarian cancer patients. [file 13148_2019_748_MOESM1_ESM.tif]
