# Supplementary material for: Hypermethylation of mismatch repair gene hMSH2 associates with platinum-resistant disease in epithelial ovarian cancer
Source: Clin Epigenetics. 2019 Oct 30;11:153. doi: 10.1186/s13148-019-0748-4 (PMC6822346; doi:10.1186/s13148-019-0748-4)
Supplement: Supplementary file 2 — Additional file 2: Table S1. Clinicopathological information for the discovery cohort patients with ovarian cancer. [file 13148_2019_748_MOESM2_ESM.docx]

**Supplementary Table S1 Clinicopathological information for the discovery cohort patients with ovarian cancer**

| **Patients num.** | **FIGO^a^ stage** | **Histology** | **Grade** | **Chemotherapy** | **PFS** | **Sensitive/Re-sistant** |
| --- | --- | --- | --- | --- | --- | --- |
| T2a | IIIc | SOC^b^ | G3 | Carboplatin, Taxol | 25 | S |
| T3a | IVa | SOC | G2 | Carboplatin, Taxol | 27 | S |
| T4a | IIIb | SOC | G1 | Cisplatin, Taxol | 30 | S |
| T5a | IIIc | SOC | G1 | Carboplatin, Taxol | 33 | S |
| T6a | IVa | SOC | G2 | Cisplatin, Taxol | 30 | S |
| T7a | IIIc | SOC | G3 | Carboplatin, Taxol | 26 | S |
| T8a | IIIa | SOC | G3 | Carboplatin, Taxol | 31 | S |
| T9a | IIIc | SOC | G3 | Cisplatin, Taxol | 27 | S |
| T2b | IIIc | SOC | G2 | Carboplatin, Taxol | 3 | R |
| T3b | IIIb | SOC | G3 | Carboplatin, Taxol | 4 | R |
| T4b | IVb | SOC | G1 | Carboplatin, Taxol | 3 | R |
| T5b | IVa | SOC | G3 | Cisplatin, Taxol | 4 | R |
| T6b | IIIc | SOC | G1 | Cisplatin, Taxol | 5 | R |
| T7b | IIIc | SOC | G2 | Carboplatin, Taxol | 2 | R |
| T8b | IIIc | SOC | G3 | Carboplatin, Taxol | 3 | R |
| T9b | IIIb | SOC | G3 | Cisplatin, Taxol | 4 | R |

^a^FIGO: International Federation of Gynecology and Obstetrics

^b^SOC: Serous ovarian cancer
